# Purified TPC Isoforms Form NAADP Receptors with Distinct Roles for Ca^2+^ Signaling and Endolysosomal Trafficking

**DOI:** 10.1016/j.cub.2010.02.049

**Published:** 2010-04-27

**Authors:** Margarida Ruas, Katja Rietdorf, Abdelilah Arredouani, Lianne C. Davis, Emyr Lloyd-Evans, Heidi Koegel, Timothy M. Funnell, Anthony J. Morgan, John A. Ward, Keiko Watanabe, Xiaotong Cheng, Grant C. Churchill, Michael X. Zhu, Frances M. Platt, Gary M. Wessel, John Parrington, Antony Galione

**Affiliations:** 1 Department of Pharmacology, University of Oxford, Mansfield Road, Oxford OX1 3QT, UK; 2 Department of Neuroscience and Center for Molecular Neurobiology, The Ohio State University, 1060 Carmack Road, Columbus, OH 43210, USA; 3 Department of Molecular Biology, Cellular Biology and Biochemistry, Brown University, 185 Meeting Street, Box G-L173, Providence, RI 02912, USA

**Keywords:** CELLBIO, SIGNALING

## Abstract

Intracellular Ca^2+^ signals constitute key elements in signal transduction. Of the three major Ca^2+^ mobilizing messengers described, the most potent, nicotinic acid adenine dinucleotide phosphate (NAADP) is the least well understood in terms of its molecular targets [1]. Recently, we showed that heterologous expression of two-pore channel (TPC) proteins enhances NAADP-induced Ca^2+^ release, whereas the NAADP response was abolished in pancreatic beta cells from *Tpcn2* gene knockout mice [2]. However, whether TPCs constitute native NAADP receptors is unclear. Here we show that immunopurified endogenous TPC complexes possess the hallmark properties ascribed to NAADP receptors, including nanomolar ligand affinity [3–5]. Our study also reveals important functional differences between the three TPC isoforms. Thus, TPC1 and TPC2 both mediate NAADP-induced Ca^2+^ release, but the subsequent amplification of this trigger Ca^2+^ by IP_3_Rs is more tightly coupled for TPC2. In contrast, TPC3 expression suppressed NAADP-induced Ca^2+^ release. Finally, increased TPC expression has dramatic and contrasting effects on endolysosomal structures and dynamics, implicating a role for NAADP in the regulation of vesicular trafficking. We propose that NAADP regulates endolysosomal Ca^2+^ storage and release via TPCs and coordinates endoplasmic reticulum Ca^2+^ release in a role that impacts on Ca^2+^ signaling in health and disease [6].

## Highlights

► Endogenous TPC protein complex has hallmark properties of native NAADP receptors ► TPCs differentially localize to subcellular sites of NAADP-induced Ca^2+^ release ► Different TPC isoforms show differential roles in NAADP-evoked Ca^2+^ release ► Altered TPC expression has dramatic, differential effects on endolysosomal function

## Results and Discussion

The identity of the NAADP receptor has long been debated [1]. Recently we demonstrated a central role for two-pore channels (TPCs) in the NAADP response [2], but whether they constitute the actual NAADP receptor remains unresolved. Here we directly examined this issue and performed the first systematic characterization of all three TPC isoforms from sea urchin, in whose eggs NAADP was first identified as a Ca^2+^ mobilizing messenger [7] and which is now considered the gold standard system for NAADP receptor studies [8].

### Sea Urchins Express Three TPC Isoforms

**Figure 1:**
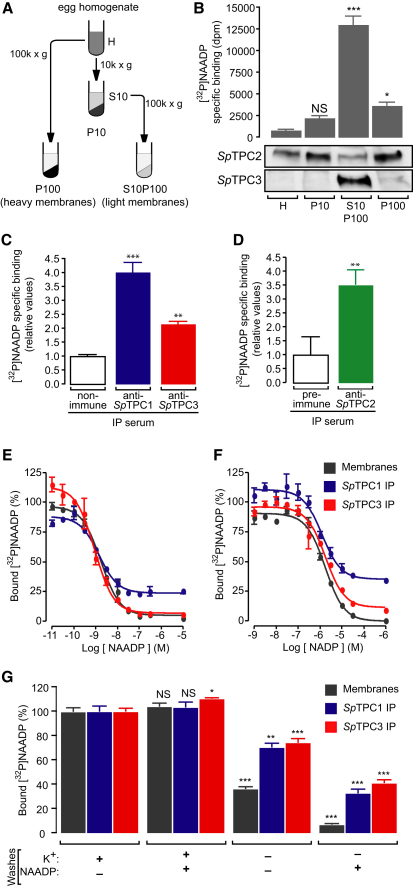
Endogenous *Sp*TPC Protein Complexes Bind to [^32^P]NAADP with Hallmark Properties of the NAADP Receptor (A) Schematic representation of protocol used for membrane preparations from *Strongylocentrotus purpuratus* egg homogenates via differential centrifugation. The following abbreviations are used: H, homogenate; S, supernatant; P, pellet. (B) Levels of specific [^32^P]NAADP binding in membrane preparations (n = 3). Panels below graph correspond to immunoblots probed with affinity-purified *Sp*TPC isoform-specific antibodies showing high molecular weight forms of *Sp*TPCs. (C) Specific [^32^P]NAADP binding for *Sp*TPC1 and *Sp*TPC3 immunoprecipitates (IPs) from S10P100 fractions with anti-*Sp*TPC1 and anti-*Sp*TPC3 sera. Values (n = 6) determined by Cerenkov counting were normalized against control IP with nonimmune serum. (D) Specific [^32^P]NAADP binding for *Sp*TPC2 IP from P100 fractions with anti-*Sp*TPC2 serum. Values (n = 11) obtained by phosphorimaging were normalized against control IP with preimmune serum. (E) Inhibition of [^32^P]NAADP binding to S10P100 membranes or *Sp*TPC1 or *Sp*TPC3 IPs by increasing concentrations of NAADP (n = 3). Values were normalized to amount of bound [^32^P]NAADP in absence of competitor. (F) Same as in (E), but with NADP as competitor (n = 3). (G) Bound [^32^P]NAADP to S10P100 membranes or *Sp*TPC1 or *Sp*TPC3 IPs after washes with buffer with K^+^ (GluIM) or without K^+^ (HEPES), in the absence or presence of 10 μM NAADP. Values (n = 3) of remaining radioactivity were normalized to values obtained for GluIM washes in the absence of NAADP. Data are represented as mean ± standard error of the mean (SEM); p > 0.05 (not significant, NS), ^∗^p < 0.05, ^∗∗^p < 0.01, ^∗∗∗^p < 0.001. See also Figure S1.

We screened the *Strongylocentrotus purpuratus* (*Sp*) genome [9] and identified three distinct isoforms, *Sp*TPC1, *Sp*TPC2, and *Sp*TPC3, which we cloned from urchin ovary. Their overall homology at amino acid level is between 29% and 34%. We generated polyclonal antibodies against each *Sp*TPC and assessed specificity by immunoblotting (see Figure S1A available online) and immunoprecipitation (Figure S1B) of membrane proteins from HEK293 cells expressing each HA.*Sp*TPC. The antibodies recognized endogenous *Sp*TPC2 in urchin egg membrane preparations, mainly in heavy fractions (P100), containing lysosome-related reserve granules [10], and recognized *Sp*TPC3 in lower-density fractions (S10P100) (Figures 1A and 1B; Figure S1C), which correlates with the highest specific [^32^P]NAADP binding (Figure 1B). *Sp*TPC2 and *Sp*TPC3 run predominantly as high molecular weight forms resistant to reducing agents (Figure S1C), possibly as tightly associated homodimers, as observed for heterologously expressed *Mm*TPC2 [11]. In line with previous observations that mammalian TPCs are glycosylated [2, 11], both endogenous and heterologously expressed *Sp*TPC2 are modified by N-glycosylation (Figures S1C and S1D); however, no N-glycosylation was detected for endogenous *Sp*TPC3 (data not shown) or heterologously expressed *Sp*TPC1 and *Sp*TPC3 (Figure S1D). Lack of antibodies suitable for immunoblotting prevented analysis of endogenous *Sp*TPC1 protein.

### Immunopurified Endogenous TPC Complexes Possess the Hallmark Properties of Native NAADP Receptors

Cell membranes expressing recombinant *Hs*TPC2 have enhanced [^32^P]NAADP binding [2]. To ascertain whether TPCs are part of the endogenous NAADP-binding complex, we immunoprecipitated endogenous *Sp*TPC proteins from solubilized egg membranes: *Sp*TPC2 from P100, and *Sp*TPC1 and *Sp*TPC3 from S10P100 fractions. All three *Sp*TPC immunoprecipitates were associated with significantly greater [^32^P]NAADP binding than controls (Figures 1C and 1D), with no such associated binding for non-sea urchin control lysate (Figure S1E); moreover, binding was blocked by preincubation with specific antigenic peptides, but not by unrelated peptides (Figures S1F and S1G).

To establish whether the recovered binding resembled that of native NAADP receptors, we carried out NAADP competition experiments and recovered typical specific binding levels of 1000–2000 dpm for *Sp*TPC1 and 600–1000 dpm for *Sp*TPC3, revealing IC_50_s of 1.4 nM (95% confidence interval: 0.9–2.0 nM) and 0.9 nM (95% confidence interval: 0.6–1.3 nM), respectively, and precisely overlapping with the 1.7 nM (95% confidence interval: 1.3–2.3 nM) value for native membranes (Figure 1E). In contrast, binding was only competed by NADP at 1000× greater concentrations (Figure 1F), as observed in egg membranes [3, 4], likely because of trace NAADP contamination [7]. K^+^-dependent irreversible binding of [^32^P]NAADP is characteristic of NAADP binding in egg membranes [5]; similarly, binding of [^32^P]NAADP to *Sp*TPC1 and *Sp*TPC3 immunocomplexes is essentially irreversible in K^+^-rich GluIM buffer, whereas in K^+^-free media, bound [^32^P]NAADP dissociates, and dissociation is enhanced by unlabelled NAADP (Figure 1G). That endogenous TPC complexes possess these unique properties provides the first direct evidence that TPCs are integral components of endogenous NAADP receptors.

### Different TPC Isoforms Show Differential Roles in NAADP-Evoked Ca^2+^ Release

**Figure 2:**
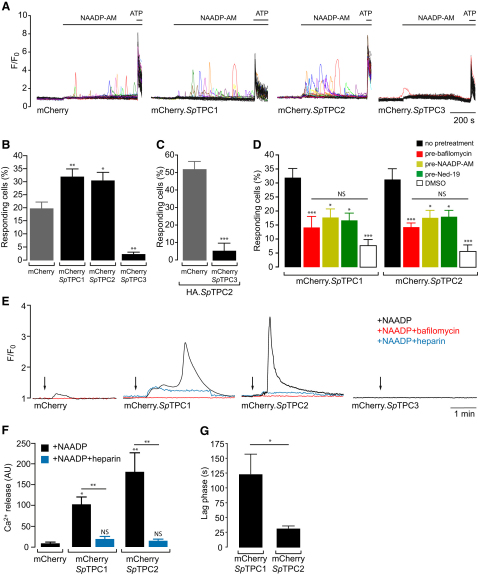
NAADP-Mediated Ca^2+^ Release in Mammalian Cells Expressing *Sp*TPCs (A) Ca^2+^ traces of mCherry.*Sp*TPC-expressing cells stimulated by 1 μM NAADP-AM. ATP (100 μM) was added at the end of each experiment to assess cell viability. The number of cells not responding to ATP was negligible. Traces of cells responding to NAADP-AM (F/F_0_ > 1.5) are shown in color. (B) Summary of results from experiments performed over 4 days with 12–22 coverslips per cell line, as outlined in (A). Only cells responding to ATP were included in the data analysis. (C) Effect of mCherry.*Sp*TPC3 transient expression on cells stably expressing HA.*Sp*TPC2. Transfection of mCherry alone is included as control. Summary of results is from experiments performed over 2 days with 4–6 coverslips per transfection. There were no significant differences between untransfected cells and cells transfected with mCherry alone (data not shown). (D) Effect of pretreatment with NAADP-AM (1 nM, 30 min), bafilomycin A1 (3 μM, 60 min), or *trans* Ned-19 (10 μM, 15 min) upon 1 μM NAADP-AM-induced Ca^2+^ release in cells expressing mCherry.*Sp*TPC1 or mCherry.*Sp*TPC2. Spontaneous activity was determined by addition of dimethyl sulfoxide (DMSO). Results from 2–3 days of experiments with 6–17 coverslips for each treatment and cell line are shown. (E) Representative Ca^2+^ traces of cells dialyzed with NAADP (100 nM) via patch pipettes in whole-cell configuration, in the absence or presence of bafilomycin A1 (1 μM) or heparin (200 μg/ml). Arrows indicate break-in. In total, 5–7 cells per condition and cell line were analyzed, and all of the cells apart from *Sp*TPC3 were responsive to NAADP dialysis, with *Sp*TPC1 and *Sp*TPC2 giving a biphasic response. (F) Summary of results from (E), quantifying total Ca^2+^ release as area under curve. (G) Summary of results from (E), quantifying the time required to trigger the secondary Ca^2+^ response after break-in. Data are represented as mean ± SEM; p > 0.05 (NS), ^∗^p < 0.05, ^∗∗^p < 0.01, ^∗∗∗^p < 0.001. See also Figure S2.

Recently, we showed that *Hs*TPC2 and *Hs*TPC1 can mediate enhanced NAADP responses in transfected cells [2]. This was subsequently confirmed for *Hs*TPC1 [12] and *Mm*TPC2, but not for *Mm*TPC1 [11]. To clarify the situation and investigate the role of TPC3, we systematically compared all three *Sp*TPC isoforms' capacity to mediate NAADP-evoked Ca^2+^ responses. HEK293 cells expressing mCherry.*Sp*TPCs were assessed for responsiveness to either NAADP dialyzed through whole-cell patch or a membrane-permeant NAADP analog (NAADP-AM) [13]. NAADP microinjection was avoided because TPC expression in HEK293 cells often results in spurious Ca^2+^ transients as a result of enhanced mechanosensitivity (data not shown). NAADP-AM evoked Ca^2+^ transients in a small number of HEK293 cells expressing mCherry alone, whereas significantly more cells expressing *Sp*TPC1 and *Sp*TPC2 responded with typically a single Ca^2+^ transient (Figures 2A and 2B). In contrast, few cells expressing *Sp*TPC3 responded to NAADP-AM with, surprisingly, even endogenous responses being suppressed (Figures 2A and 2B). Similar results were obtained with cells expressing HA.*Sp*TPCs (Figure S2A). The inhibitory effect of *Sp*TPC3 is seen despite lysosomal Ca^2+^ stores remaining replete with Ca^2+^, as demonstrated by release of Ca^2+^ by either the vacuolar H^+^-ATPase inhibitor bafilomycin A1, which abrogates pH-dependent Ca^2+^ storage [6, 10], or the lysosomotropic agent glycyl-L-phenylalanine 2-naphthylamide (GPN) (Figures S2B and S2C). Moreover, pH measurements with LysoSensor Yellow/Blue dextran indicate that luminal lysosomal pH is unaltered in cells expressing *Sp*TPC3 (Figure S2D), and responses to extracellular ATP are not affected by *Sp*TPC3 (Figure 2A; Figure S2E). In addition, mCherry.*Sp*TPC3 expression, but not mCherry alone, inhibits the enhanced NAADP responses from cells expressing HA.*Sp*TPC2 (Figure 2C). Importantly, pharmacological agents that antagonize the NAADP response greatly reduced the ability of *Sp*TPC-expressing cells to respond to NAADP-AM. Thus, bafilomycin A1, low concentrations of NAADP itself (which desensitizes the NAADP response [3, 14]), and the selective NAADP receptor antagonist, Ned-19 [15], greatly reduced the responsiveness of *Sp*TPC1- and *Sp*TPC2-expressing cells to NAADP (Figure 2D). The residual response after the pharmacological agents is likely due to spontaneous activity in this system, because they are not significantly different from those obtained with dimethyl sulfoxide vehicle alone (Figure 2D).

In a different approach, NAADP was dialyzed into the cytosol via a patch pipette, allowing observation of more detailed kinetics of the Ca^2+^ response, perhaps through increased buffering by the higher affinity Ca^2+^ indicator, fura-2 [2]. Compared to controls, cells expressing *Sp*TPC1 and *Sp*TPC2 gave greater Ca^2+^ responses to NAADP that were abolished by bafilomycin A1; furthermore, the inhibitory effect of *Sp*TPC3 was also evident (Figure 2E). Importantly, whereas the NAADP response in control cells was small and monophasic, that in *Sp*TPC1- and *Sp*TPC2-expressing cells was biphasic—an initial “pacemaker” rise followed by an explosive Ca^2+^-release phase (Figures 2E and 2F), with a longer initial Ca^2+^ release for *Sp*TPC1 than *Sp*TPC2 (Figures 2E and 2G). Application of the IP_3_R antagonist heparin via the patch pipette selectively blocked the second Ca^2+^ release phase, thus unmasking an initial NAADP trigger (Figures 2E and 2F). Importantly, responses to IP_3_ itself were identical between control cells and those expressing *Sp*TPCs (Figure S2F); thus, the effects of NAADP in *Sp*TPC-expressing cells cannot be ascribed to enhanced IP_3_ sensitivity of IP_3_Rs.

All three TPCs thus appear to play key roles in NAADP-evoked Ca^2+^ release but in distinct ways, possibly reflecting important functional differences between the NAADP receptors formed by each isoform, as well as differences in coupling to other cellular Ca^2+^-release mechanisms. Because with NAADP-AM (Figures 2A–2D), responses are generally all or nothing, it is likely that only Ca^2+^ responses amplified by IP_3_R recruitment are detected. Therefore, expression of different *Sp*TPC isoforms affects responsiveness of the cells rather than amplitude of the response. Our data thus confirm TPCs as fundamental for local NAADP-induced Ca^2+^ signals that then trigger regenerative Ca^2+^ responses via Ca^2+^-induced Ca^2+^ release mechanisms, as previously predicted from pharmacological evidence [16, 17]. The looser coupling between *Sp*TPC1 and IP_3_Rs compared to *Sp*TPC2 and IP_3_Rs may reflect the differential localization of *Sp*TPC1 and *Sp*TPC2 outlined below. In contrast, *Sp*TPC3 expression suppresses activity of endogenous TPCs, perhaps by enhancing the type 1 self-inactivation properties of the NAADP receptor [8] previously observed in urchin eggs [3, 14, 18]. Interestingly, in the mammalian systems where type 2 self-inactivation is observed [8], TPC3 is not present. However, the exact physiological role of TPC3 in different species needs further investigation, because a recent study of a different sequence variant of *Sp*TPC3 failed to detect differences between the three TPCs [19]. One of the peculiarities of sea urchin is the expression of multiple polymorphic forms of the same protein [9, 20, 21], and it is possible that naturally occurring *Sp*TPC3 sequence variants differ in their responses to NAADP.

### TPCs Differentially Localize to Subcellular Sites of NAADP-Induced Ca^2+^ Release

**Figure 3:**
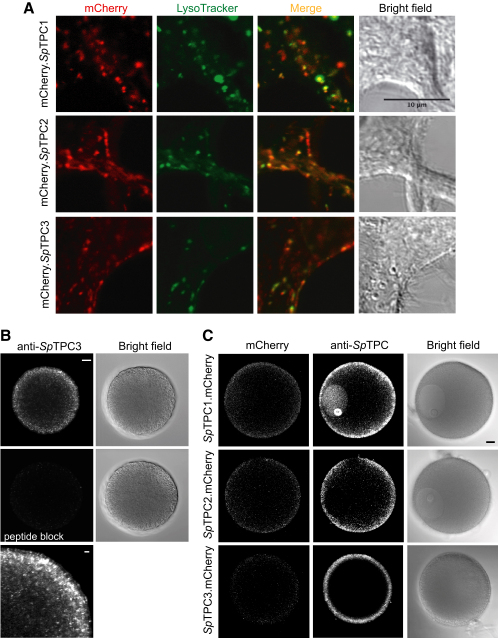
Intracellular Localization of *Sp*TPCs (A) Colocalization of mCherry.*Sp*TPC with acidic organelles (LysoTracker Green) in live HEK293 cells expressing mCherry.*Sp*TPCs. Scale bar corresponds to 10 μm. (B) Anti-*Sp*TPC3 immunofluorescence of *S. purpuratus* eggs with or without peptide block for assessment of immunostaining specificity. Scale bar corresponds to 10 μm, or 2 μm for higher magnification panel. (C) Localization of *Sp*TPCs.mCherry expressed in oocytes of starfish *Asterina miniata* and detected by mCherry fluorescence or by immunofluorescence with corresponding anti-*Sp*TPC antibodies. Scale bar corresponds to 20 μm. Noninjected controls were not labeled with anti-*Sp*TPC antibodies (data not shown). In oocytes expressing *Sp*TPC3.mCherry, the germinal vesicle is out of the plane of focus. mCherry.*Sp*TPCs showed similar cortical localization (data not shown). Oocytes expressing mCherry alone showed a signal across the entire cell (data not shown). Of note is the presence of an *Sp*TPC1 signal in the nucleus. Indeed, *Sp*TPC1 has a putative nuclear localization signal (amino acids 327–334). Its physiological significance may represent an important avenue for further analysis of TPC function, especially in light of reports describing NAADP signaling in nucleus [33, 34]. See also Figure S3.

Several lines of evidence have suggested the presence of NAADP receptors in acidic organelles [10, 22–24], including endosomes [25]. We recently showed that TPCs are associated with acidic organelles in HEK293 cells [2], confirmed in subsequent studies [11, 12]. To examine the localization patterns of each isoform, we first studied heterologously expressed mCherry-tagged *Sp*TPC1, *Sp*TPC2, and *Sp*TPC3 and observed their association with acidic organelles in live HEK293 cells, as confirmed by LysoTracker Green colocalization (Figure 3A). In addition, *Sp*TPC2 and *Sp*TPC3 (either mCherry- or HA-tagged versions) colocalize with LAMP-2 (a late endosome and lysosome marker) in fixed cells and also show some colocalization with a recycling endosome marker (transferrin receptor) (Figure S3). Similarly, mCherry.*Sp*TPC1 shows some overlap with LAMP-2 staining (Figure S3). Degrees of overlap underscore the dynamic nature of the endolysosomal system, involving hybridizing fusion events [26, 27] and motility of GFP-tagged *Hs*TPC1 and *Hs*TPC2 [2]. Our findings thus highlight important differences in the major distributions of different *Sp*TPC isoforms that may reflect distinctive functional roles.

Immunostaining of endogenous *Sp*TPC3 in urchin eggs revealed a punctate cortical staining pattern, in addition to some localization in deeper puncta (Figure 3B). Because *Sp*TPC1 and *Sp*TPC2 immunostaining was not blocked by immunogenic peptides (data not shown), we also expressed all three mCherry.*Sp*TPCs in starfish oocytes, an echinoderm system suited to heterologous expression [28]. Each *Sp*TPC.mCherry localized to the cortex and intracellular puncta (Figure 3C). This cortical distribution agrees with the cortex exhibiting greatest sensitivity to NAADP in urchin eggs [17, 29] and starfish oocytes [30], thus demonstrating for the first time that endogenous TPCs localize to subcellular loci of NAADP-induced Ca^2+^ release.

### Altering TPC Expression Has Dramatic, Differential Effects upon Endolysosomal Transport and Functions

**Figure 4:**
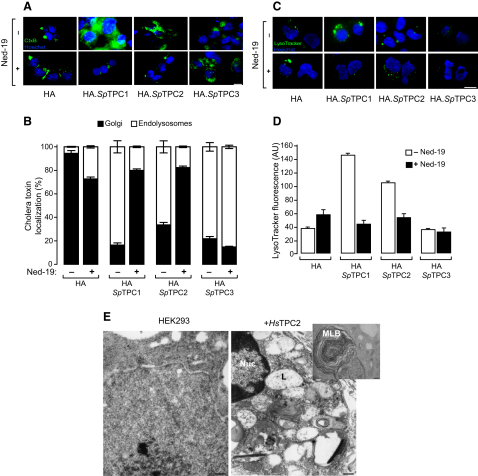
Two-Pore Channel Overexpression Causes Changes in Endolysosomal Trafficking and Morphology (A) Lipid endocytosis and recycling in HA.*Sp*TPC-overexpressing HEK293 cells assessed by Alexa Fluor 568-cholera toxin B subunit (CtxB; green). Nuclei are labeled with Hoechst 33342 (Hoechst, blue). Scale bar corresponds to 5 μm. Endolysosomal localization was confirmed by colocalization with endocytosed high molecular weight rhodamine dextran (data not shown); Golgi localization by a single perinuclear distribution is not overlapping with the dextran. (B) Summary of results from experiments in (A) corresponding to three separate experiments with a minimum of 50 cells analyzed per experiment. (C) Pattern of lysosomal staining by LysoTracker Green (LysoTracker, green) in HA.*Sp*TPC-overexpressing HEK293 cells, in the absence or presence of NAADP receptor antagonist Ned-19 (10 μM, 12 hr). Nuclei were labeled with Hoechst 33342 (Hoechst, blue). Scale bars correspond to 5 μm. (D) Summary of results from experiments in (C) corresponding to three separate experiments, with a minimum of 50 cells analyzed per experiment. To allow meaningful comparison between images, we standardized LysoTracker loading conditions and imaging protocols for all cell types. pH differences between cell types were negligible, as assessed by fluorescein-dextran fluorescence (data not shown). (E) Lysosomal storage disease phenotype in HA.*Hs*TPC2-overexpressing HEK293 cells visualized by electron microscopy. Inset is a magnification of one region of a HA.*Hs*TPC2-overexpressing cell. Scale bars correspond to 200 nm. The following abbreviations are used: Nuc, nucleus; MLB, multiple lamellar inclusion body; L, lysosome. Data are represented as mean ± SEM. See also Figure S4.

Given the importance of Ca^2+^ signals for endolysosomal function [26, 27], we investigated the effect of altering TPC expression upon cellular trafficking. We assessed endolysosomal transport via the fate of Alexa Fluor 594-conjugated cholera toxin B subunit, which, upon binding to plasma membrane ganglioside GM1, is normally internalized and delivered to the Golgi via early endosomes [6]. Although control cells expressing HA alone accumulate cholera toxin in the Golgi, cells expressing HA.*Sp*TPCs retain cholera toxin within endolysosomes (Figures 4A and 4B), with *Sp*TPC1 cells showing the severest dysfunction. Although *Sp*TPC3-expressing cells also fail to accumulate cholera toxin within the Golgi, their tighter pattern of accumulation close to the nucleus suggests a transport block at a different point during endocytosis. These phenotypes indicate generalized and fundamental endocytic dysfunction characteristic of many lysosomal storage disorders [31]. Indeed, overexpression of either *Sp*TPC1 or *Sp*TPC2, but not of *Sp*TPC3 (Figures 4C and 4D), led to dramatically enlarged lysosomes. The observed phenotypes are not simply due to overexpression of lysosomal proteins, because heterologous expression of LAMP-1 or LAMP-2 does not affect endolysosomal trafficking [32].

Following 12 hr treatment with the NAADP receptor antagonist Ned-19 [15], cells overexpressing *Sp*TPC1 or *Sp*TPC2 showed normalization of both cholera toxin trafficking to the Golgi (Figures 4A and 4B) and lysosomal size and distribution (Figures 4C and 4D). In contrast, Ned-19 had no effect upon cells overexpressing *Sp*TPC3 (Figures 4A–4D). Furthermore, in control cells, Ned-19 caused a partial inhibition of cholera toxin trafficking (Figures 4A and 4B) and a slight elevation in LysoTracker Green accumulation (Figures 4C and 4D), consistent with a role for NAADP signaling in normal endolysosomal function.

These data support a role for NAADP and TPCs in normal endolysosomal function, perhaps by allowing local Ca^2+^ signals evoked by TPC activation to in turn regulate lysosomal biogenesis and other endolysosomal processes [27] (Figure S4), with both reduced or excessive TPC activity leading to deregulation (Figure 4).

Interestingly, two human lysosomal diseases, Niemann-Pick disease [6] and mucolipidosis IV [26], involve deregulated Ca^2+^ homeostasis. In Niemann-Pick, Ca^2+^ storage and NAADP-evoked Ca^2+^ release are reduced [6], whereas in mucolipidosis IV, they are enhanced (data not shown). The cellular pathologies induced by TPC overexpression mirror the complex morphological endolysosomal characteristics of these diseases. Electron microscopy analysis confirms that most *Hs*TPC2-overexpressing cells (87%) show conspicuous heterogeneous multiple lamellar inclusion bodies (Figure 4E), indicating generalized endocytic dysfunction and storage of multiple lipid species resembling lysosomal storage disorder cells [31], implicating a possible role for TPC dysfunction in these diseases.

### Conclusions

Our findings confirm TPCs as integral components of endogenous NAADP receptors, demonstrate that TPCs are both functionally and spatially diverse endolysosomal channels, and show that perturbation of TPC function and NAADP signaling might both underlie, and be used to ameliorate, disorders of endolysosomal trafficking.

## Supplemental Information

Document S1. Supplemental Experimental Procedures and Four Figures
