# Supplementary material for: Purified TPC Isoforms Form NAADP Receptors with Distinct Roles for Ca2+ Signaling and Endolysosomal Trafficking
Source: Curr Biol. 2010 Apr 27;20(8-6):703–9. doi: 10.1016/j.cub.2010.02.049 (PMC2861162; doi:10.1016/j.cub.2010.02.049)
Supplement: Document S1. Supplemental Experimental Procedures and Four Figures [file mmc1.pdf]

Current Biology, Volume *20*

Supplemental Information

Purified TPC Isoforms Form NAADP Receptors with Distinct Roles for Ca^{2+} Signaling and Endolysosomal Trafficking

Margarida Ruas, Katja Rietdorf, Abdelilah Arredouani, Lianne C. Davis, Emyr Lloyd-Evans, Heidi Koegel, Timothy M. Funnell, Anthony J. Morgan, John A. Ward, Keiko Watanabe, Xiaotong Cheng, Grant C. Churchill, Michael X. Zhu, Frances M. Platt, Gary M. Wessel, John Parrington, and Antony Galione

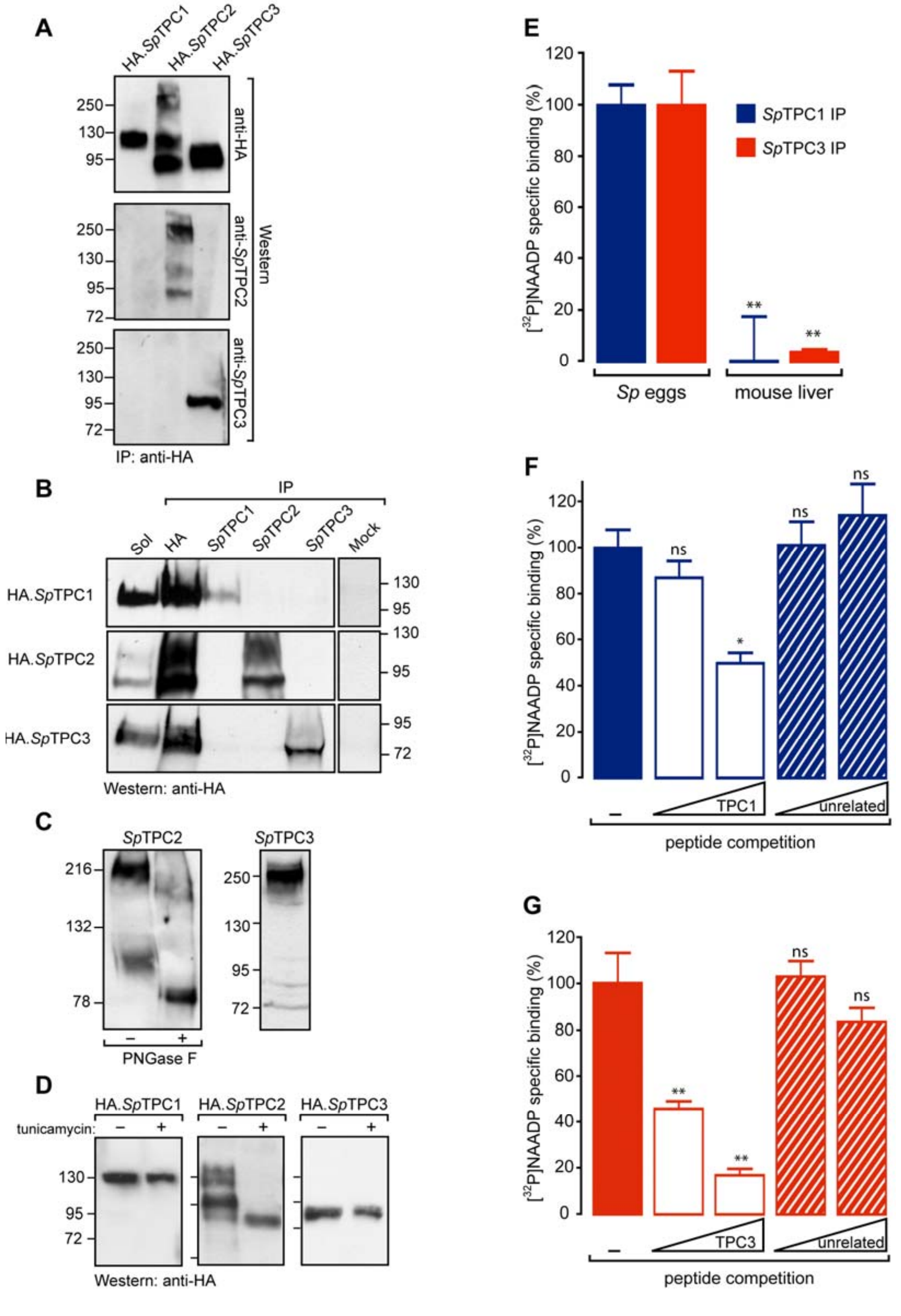

Figure S1. Immunoblotting and Immunoprecipitation of *Sp*TPC Complexes

(A) Characterization of anti-*Sp*TPC antibodies by immunoblotting. Anti-HA IPs from lysates of HEK293 cells expressing HA.*Sp*TPCs were probed with anti-*Sp*TPC purified antibodies or anti-HA antibodies. Antibodies against *Sp*TPCs only recognized their specific isoform. The anti-

*Sp*TPC1 antibody failed to detect the corresponding HA.*Sp*TPC1 protein in immunoblots (data not shown).

(B) Characterization of anti-*Sp*TPC antibodies by immunoprecipitation. Anti-*Sp*TPC antibodies were able to immunoprecipitate (IP) the corresponding proteins from HEK293 cells expressing HA.*Sp*TPCs prepared in the same buffer as that used for the IP-binding assays. All antibodies used showed specificity towards their target isoform. Anti-HA IP was included as a positive control. Non-immune rabbit serum (Mock) fails to IP HA.*Sp*TPCs present in the solubilized sample (Sol). IPs were immunoblotted with an anti-HA antibody.

(C) Immunoblots of membrane fractions of *S. purpuratus* egg homogenates, with or without PNGase F treatment (24 h), probed with anti-*Sp*TPC2 or anti-*Sp*TPC3 purified antibodies. Both *Sp*TPC2 and *Sp*TPC3 are present mainly as high molecular weight forms resistant to reducing agents (200 mM DTT). *Sp*TPC2 is a glycoprotein as shown by a decrease of its apparent molecular weight upon N-glycosidase treatment.

(D) Anti-HA immunoblots of lysates from HEK293 cells expressing HA.*Sp*TPCs grown in the presence or absence of tunicamycin (1 µg/ml). Treatment of cells (24 h) with this N-glycosylation inhibitor results in the appearance of lower molecular weight non-glycosylated forms of *Sp*TPC2. No N-glycosylation was detected for *Sp*TPC1 or *Sp*TPC3 in this system.

(E) [^{32}P]NAADP-binding associated with anti-*Sp*TPC IPs is dependent on the presence of corresponding *Sp*TPC, as IPs from a solubilized mouse liver sample do not bind [^{32}P]NAADP. Values were normalized against specific [^{32}P]NAADP binding recovered from IPs using solubilized *S. purpuratus* (*Sp*) S10P100 sample (n=3).

(F) [^{32}P]NAADP binding associated with *Sp*TPC1 IP using specific sera from immunized rabbits is decreased in samples where specific peptides were used for inhibition of *Sp*TPC protein binding to antibodies, but unaffected by incubation with unrelated peptides. Peptides were used at 10 µg/ml and 100 µg/ml. Values were normalized against [^{32}P]NAADP binding obtained in non-competition controls (n=3).

(G) Same as in (F) using anti-*Sp*TPC3 specific serum and corresponding peptides (n=3).

Numbers on the left of immunoblots correspond to the position of molecular weight markers (kDa).

Data are represented as mean ± SEM. $p>0.05$ (ns), $p < 0.05$ (*), $p<0.01$ (**).

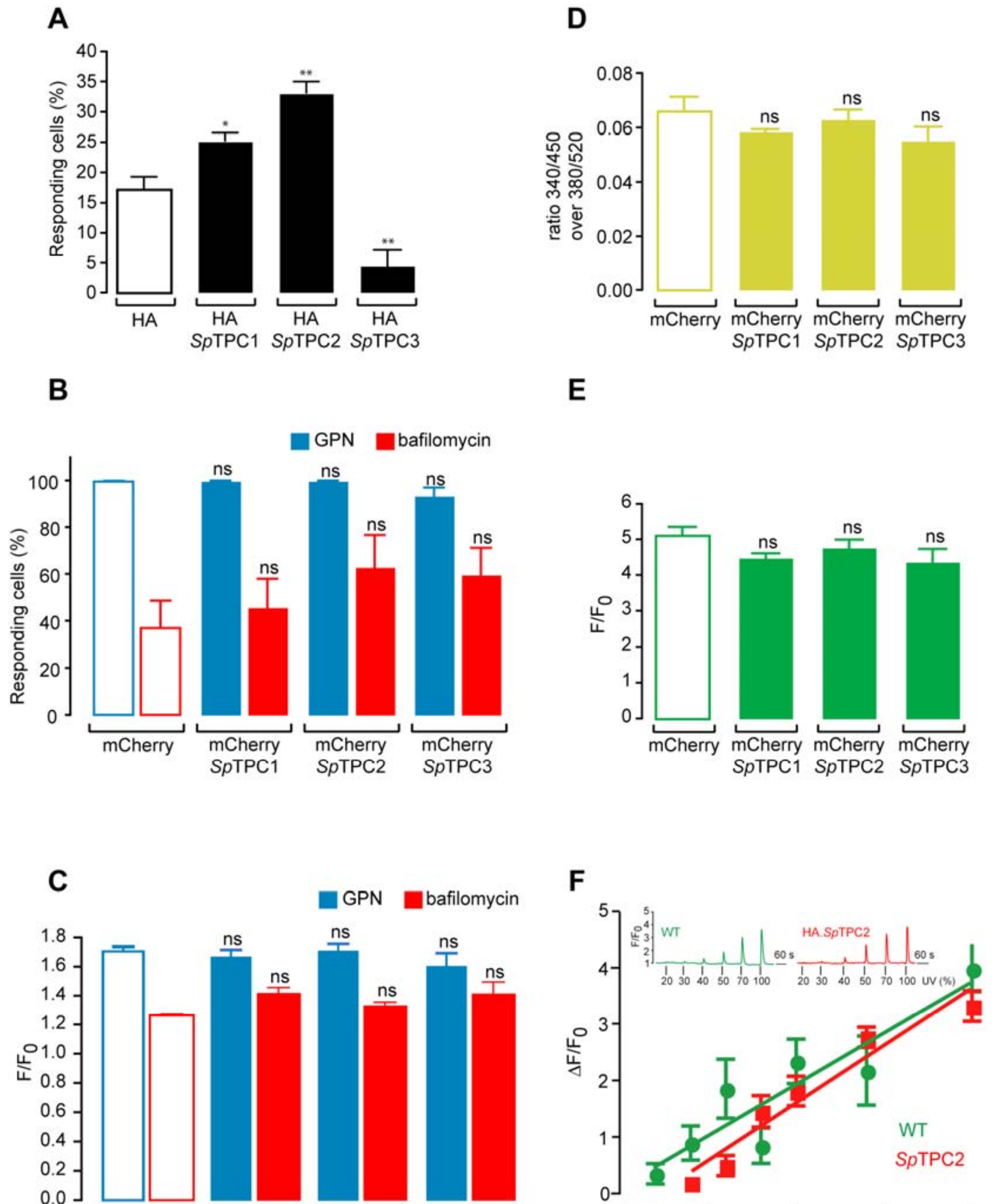

Figure S2. Controls for Ca^{2+} Responses in HEK293 Cells Expressing *Sp*TPCs

(A) Summary of results obtained from Ca^{2+} traces, upon addition of 1 µM NAADP-AM to cells expressing HA.*Sp*TPCs. Only cells showing a transient increase in the normalized fluo-5F fluorescence F/F_0 > 1.5 were considered as responding cells. Experiments were performed over 3 days with 4-8 coverslips for each cell line.

(B,C) Summary of results obtained from Ca^{2+} traces, upon addition of 200 µM GPN or 3 µM bafilomycin A1 to cells expressing mCherry.*Sp*TPCs. Only cells showing a transient increase in the normalized fura-2 AM fluorescence F/F_0 > 1.2 were considered as responding cells. Graphs

show (B) percentage of responding cells or (C) maximum amplitude of Ca^{2+} release. Experiments were performed over 3 days with 6-7 coverslips for each cell line.

(D) Ratiometric determination of lysosomal luminal pH. Lysosomal fluorescence was measured and averaged for 4-5 different regions in different coverslips. Experiments were performed over 2 days with 6 coverslips for each cell line.

(E) Summary of results obtained from Ca^{2+} traces, upon addition of 100 µM ATP (after addition of NAADP-AM) to cells expressing mCherry.*Sp*TPCs. Experiments were performed over 4 days with 12-22 coverslips per cell line.

(F) Collated data of Ca^{2+} traces, measured with fluo-3, of HEK293 cells or HEK293 cells expressing *Sp*TPC2, upon photolysis of caged IP_3 with increasing UV laser power. Analysis was done on 7-34 cells. No responses to UV were observed in cells microinjected with fluo-3 alone (data not shown). Analysis of the linear regression lines revealed no significant difference between cell types ($P>0.2$). Even if a 2nd order polynomial fit was used instead, there was still no significant difference between the two groups (data not shown). Inset shows representative Ca^{2+} traces. Note that the laser power sequence was randomized from run to run but we show the traces from matching protocols.

Data are represented as mean ± SEM and statistical analysis done against control cells expressing mCherry or HA. $p>0.05$ (ns), $p < 0.05$ (*), $p<0.01$ (**).

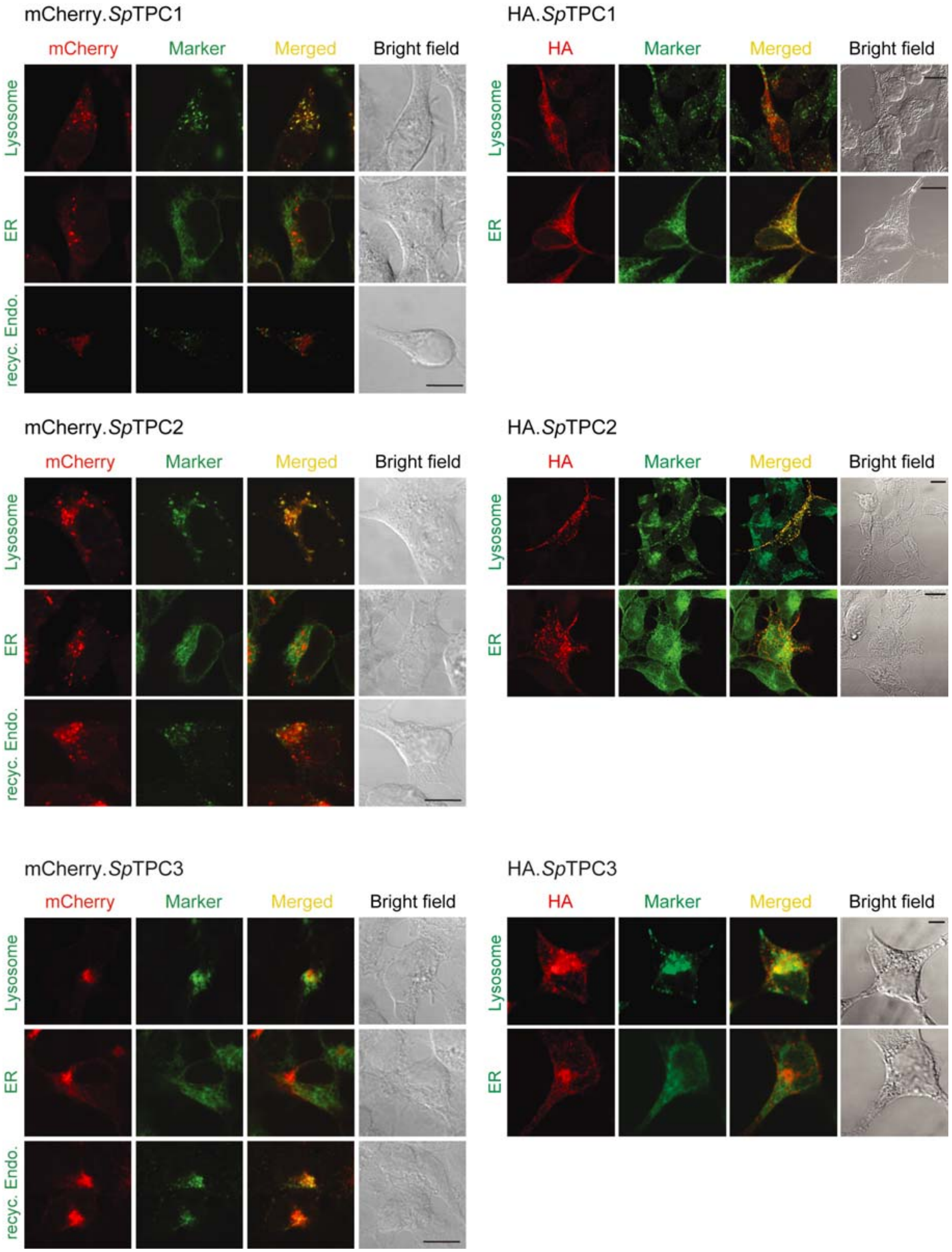

Figure S3. Colocalization of *Sp*TPCs in HEK293 Cells with Organelle Markers

HEK293 cells expressing mCherry.*Sp*TPCs or HA.*Sp*TPCs were fixed, permeabilized and immunostained with different organelle markers (see Supplemental Experimental Procedures) for assessment of co-localization. Scale bar corresponds to 10 µm. No co-localization was detected for mitochondrial or Golgi markers (data not shown). Control experiments showed no cross-species reactivity between antibodies used (data not shown).

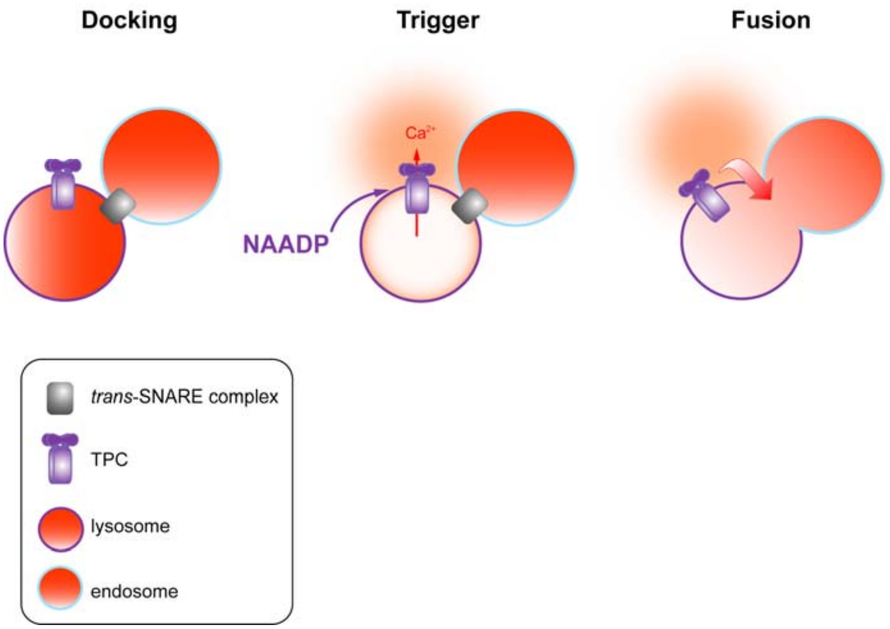

Figure S4. Possible Role of TPCs in Endolysosomal Fusion Events

Schematic representation of a model outlining how NAADP and TPCs might provide Ca^{2+} to drive vesicle fusion. The high [Ca^{2+}] within the lumen of endosomes and lysosomes is represented by the red fill. NAADP gates the TPC Ca^{2+}-release channels residing in the vesicle membrane to locally elevate the cytosolic [Ca^{2+}]. This Ca^{2+} then acts in an 'autocrine' manner to promote fusion of organelles already docked by the trans-SNARE complex [1, 2]. For simplicity, we have depicted heterotypic fusion between endosomes and lysosomes and with TPCs on only one of the fusion partners; clearly, there are several other possible arrangements not depicted here such as homotypic fusion (endosome-endosome, lysosome-lysosome) as well as TPCs being present on both fusion partners (possibly different isoforms).

Supplemental Experimental Procedures

Cloning *S. purpuratus* TPC cDNAs

*Sp*TPC cDNAs were cloned from ovary or testis RNA as previously described [3]. Full-length cDNAs were obtained from ovary using the following primers:
*Sp*TPC2 (F: ATGGGAGACTACTACGAGTATGAG; R: TTAGAAGCGCAGGTTTTGTAT),
*Sp*TPC3 (F: ATGGAGGGGCCAAAGGATTAT; R: TGGATGAAACACACACATTCG)
Full-length *Sp*TPC1 cDNA was obtained by ligating 3 overlapping clones using internal restriction sites:
clone 1 (F: AAATCTCGCAAATTCTCGCC; R: CAAAGGTCGCTTTACCTGGA),
clone 2 (F: AACTACAGGGAGGCGGCTAT; R: TCGGCTTCCTTGATCCATT),
clone 3 (F: AACATCTTTGAGGTGTCGAGG; R: AGCCTTTTGTACCCACATGA).
The following reaction parameters were used: 50 ºC (30 min); 94 ºC (2 min); 40 cycles of 94 ºC (15 s), 51 ºC (30 s) and 68 ºC (3 min); final extension at 68 ºC (5 min). Several independent RT-PCR clones were sequenced and the ones used in this study deposited in GeneBank under accession numbers: EU287986 (*Sp*TPC1), EU287987 (*Sp*TPC2) and EU287988 (*Sp*TPC3)

Antibodies

Antibodies specific for *Sp*TPCs were raised commercially (Covalab or Eurogentec) by immunizing rabbits simultaneously with two specific peptides derived from the protein sequences. (*Sp*TPC1: peptide a, CEVSRLKWKSQREERL; peptide b, AYRGTRQRTKADLSKC. *Sp*TPC2: peptide a, QKQPIHRKVYPIYGC; peptide b, CDEIYKHPHIQNLRF. *Sp*TPC3: peptide a, MEGPKDYVDSYMPKSC; peptide b, TSLDKTTFSEPSSPVC) coupled to BSA. Specific antibodies against *Sp*TPCs were affinity purified from immunized rabbit serum using peptides a and b conjugated to Sulfo-Link agarose (Pierce). A rat monoclonal antibody against the HA peptide (from hemagglutinin) (clone 3F10, Roche) was used to detect tagged proteins. Organelle markers used were mouse monoclonal antibodies: anti-LAMP-2 for lysosomes (clone H4B4, Santa Cruz), anti-PDI for ER (clone RL90, Abcam), anti-GM130 for Golgi (clone 35/GM130, BD Transduction Laboratories), anti-cytochrome C for mitochondria (clone 6H2.B4, Invitrogen) and anti-transferrin receptor for recycling endosomes (clone 236-15375, Invitrogen). Secondary antibodies used for immunoblots were HRP-labelled goat anti-rabbit IgG (Sigma), and HRP-labelled goat anti-rat IgG (Sigma). Secondary antibodies for immunofluorescence were Alexa Fluor 488- and Alexa Fluor 546-labelled goat anti-rat IgG and goat anti-mouse IgG, all cross-adsorbed (Invitrogen). For immunostaining of *Strongylocentrotus purpuratus* eggs and *Asterina miniata* oocytes Cy3- and Cy5-labelled $F(ab')_2$ fragments of goat anti-rabbit IgGs (Jackson ImmunoResearch Laboratories) were used.

Mammalian Expression of TPCs

Full-length *Sp*TPC cDNAs were subcloned into pcDNA5TO (Invitrogen) to allow tetracycline-regulated expression in mammalian cells expressing the tetracycline repressor. 2xHA, or mCherry tags were placed at the 5' end of the TPC cDNA (named throughout the text as HA.*Sp*TPCs or mCherry.*Sp*TPCs, respectively). Constructs were transfected into HEK293 cells expressing the tetracycline repressor (TREx™-293, Invitrogen) using the jetPEI reagent (Qbiogene) and clones of hygromycin-resistant cells screened for inducible expression of *Sp*TPCs. Clones were propagated at 37 ºC, 5 % CO_2 in growth medium composed of DMEM, 10 % tetracycline-free foetal calf serum, 100 U/ml penicillin, 100 µg/ml streptomycin, 2 mM

glutamine, 5 µg/ml blasticidin and 100 µg/ml hygromycin B. HEK293 cells expressing HA.*Hs*TPC2 constitutively [4] were maintained in 37 ºC, 5 % CO_2 in growth medium composed of DMEM, 10 % foetal calf serum, 100 U/ml penicillin, 100 µg/ml streptomycin, 2 mM glutamine and 500 µg/ml geneticin. Where indicated cells were grown for 24 h in the presence of 1 µg/ml of tunicamycin or equivalent volume of carrier solvent (DMSO). For transient transfections of HA.*Sp*TPC2 cells with mCherry.*Sp*TPC3, cells were transfected, induced and analysed 24 h later.

Echinoderm Expression

Full-length *Sp*TPC cDNAs were subcloned into pCRII-TOPO (Invitrogen). An mCherry tag was placed at either the 5' or 3' end of the *Sp*TPC cDNA (named throughout the text as mCherry.*Sp*TPCs or *Sp*TPCs.mCherry, respectively). RNA for microinjection was transcribed from linearized pCRII-TOPO constructs using the mMessage mMachine T7 or SP6 kit (Ambion) and purified using the RNeasy micro kit (QIAGEN). Immature oocytes of the starfish, *A. miniata*, were washed in Ca^{2+}-free artificial sea water for 30-45 min before use to remove the follicle cells. Immature oocytes were placed in a 200 µm-opening Nitex mesh (SEFAR) and microinjected with 0.5 mg/ml RNA (pipette concentration) using an Eppendorf FemtoJet. The amount of injected material was estimated at 1-2 % of the oocyte volume. FITC-labelled dextran (1 mM) was coinjected into oocytes as a marker. The oocytes were incubated in artificial sea water at 15 ºC for 48 h before analysis. Signal from mCherry was visualized on a Zeiss 510 confocal microscope using the excitation/emission parameters: (561/>575 nm).

Membrane Preparation

All preparations were done in the presence of protease inhibitors (Roche). Mammalian cells were homogenized in hypotonic buffer (20 mM Hepes pH 7.2, 1 mM EDTA) and the nuclei and cell debris were removed by centrifugation at 1.5k *g* for 5 min at 4 °C. The resulting supernatant was centrifuged at 100k *g* for 60 min at 4 °C. The membrane pellet was resuspended in hypotonic buffer and aliquots stored at -80 ºC. *S. purpuratus* egg homogenates in GluIM (250 mM potassium gluconate, 250 mM N-methylglucamine, 1 mM $MgCl_2$, 20 mM HEPES, pH 7.2) [5] were used for sea urchin membrane preparations. Homogenates were diluted 1/20 in GluIM and centrifuged at 9000 *g* for 10 min. The resulting pellet was resuspended in GluIM (P10) and the supernatant was further centrifuged at 100k g for 1 h to produce a pellet (S10P100) that was also resuspended in GluIM. Sample aliquots were stored at -80 ºC. Where indicated, native membranes were treated with PNGase F (Calbiochem) at room temperature for 24 h. Control reactions were performed in similar conditions with no enzyme.

Immunoblots

Proteins were resolved by SDS-PAGE using 7% acrylamide gels and transferred to PVDF membranes (GE Healthcare). Blocked membranes were incubated with affinity-purified antibodies in PBS, 0.5 % Tween-20, and blocking agent (either 5 % dried milk or agent provided with the chemiluminescence reagents (GE Healthcare). Secondary antibodies used were anti-rat or anti-rabbit IgGs coupled to HRP (SIGMA). Immunoreactive bands were detected by chemiluminescence using ECL reagents (GE Healthcare). To minimize heat-induced protein aggregation, protein samples loaded on gels contained 200 µM DTT and were not heat treated.

Immunoprecipitation

Samples were solubilized in GluIM, 1 % CHAPS in the presence of protease inhibitors (Roche) and were subjected to immunoprecipitation by incubation with anti-HA IgG, or anti-TPC sera at 4 ºC overnight. Immunocomplexes were pulled-down by incubation with either protein G (for rat monoclonal antibodies) or protein A (for rabbit polyclonal antibodies) beads (both from GE Healthcare). For peptide inhibition, sera were pre-incubated with either specific or non-related peptides prior to immunoprecipitation at 10 µg/ml and 100 µg/ml. S10P100 fractions were used for anti-*Sp*TPC1 and *Sp*TPC3 and P100 for anti-*Sp*TPC2 immunoprecipitations.

Immunofluorescence

Cells were fixed with paraformaldehyde (4 % paraformaldehyde in PBS for mammalian cells and 2 % paraformaldehyde in artificial sea water for echinoderms) and permeabilized in 0.2 % Triton X-100 in PBS (for mammalian cells) or in methanol (for echinoderm cells). Mammalian cells were pre-blocked in PBS, 5 % (horse or goat) serum, 0.1 % Triton X-100 and echinoderm cells were pre-blocked in 10 % sheep serum, 1 % BSA before probing with affinity-purified primary antibodies. Where indicated peptide-block controls were performed using antibodies pre-adsorbed to peptides in nitrocellulose membranes. Primary antibody staining was visualized with Alexa Fluor-labelled secondary antibodies (for mammalian cells) (Invitrogen), or Cy3- and Cy5-labelled secondary antibodies (Jackson ImmunoResearch Laboratories) for echinoderm cells. Coverslips were mounted on slides using ProLong Gold Antifade Reagent (Invitrogen). Cells were viewed on a Zeiss 510 META confocal microscope, in multitrack mode to reduce bleed-through, using the following excitation/emission parameters (nm): Alexa 488 (488/505-530); mCherry, Alexa 546 (543/>560); FITC (488/>505); Cy3, mCherry (561/>575); Cy5 (633/>650).

Radioligand Binding Assays

Binding was carried out in GluIM with 0.2 nM [^{32}P]NAADP in the presence or absence of competing nucleotides for 1 h at room temperature. Unbound [^{32}P]NAADP was removed by either rapid filtration (for native membranes), or by centrifugation through microspin filters (for immunocomplexes on protein A beads). Protein samples were washed with either binding buffer or 20 mM Hepes, pH 7.2 to test the irreversibility of binding in the presence or absence of 10 µM NAADP. Bound [^{32}P]NAADP either on filters, or eluted from beads with 1 % SDS, was determined by Cerenkov counting. In some experiments radioactivity on filters was determined by phosphorimaging using a Typhoon scanner (GE Healthcare). For immunoprecipitated samples all buffers contained 1 % CHAPS.

Live Cell LysoTracker Staining

Mammalian cells expressing mCherry constructs were grown on poly-D-lysine coated coverslips. Cells were loaded with 200 nM LysoTracker Green DND-26 (Invitrogen) in a buffer containing 121 mM NaCl, 5.4 mM KCl, 0.8 mM $MgCl_2$, 2 mM $CaCl_2$, 6 mM $NaHCO_3$, 5.5 mM D-Glucose, 25 mM Hepes, pH 7.4 for 15 min at room temperature, followed by three washes. Cells were viewed with a 63x oil immersion objective on a Zeiss 510 META confocal microscope, in multitrack mode, using the following excitation/emission parameters (nm): LysoTracker Green (488/505-530), mCherry (543/>560). Where indicated, cells were incubated with 10 µM Ned-19 for 6 h prior to staining with LysoTracker Green. Cells were visualized on a Zeiss Axioskop 2 microscope fitted with a Retiga CCD camera and Metamorph imaging suite.

Trafficking

For cholera toxin uptake cells grown on coverslips were incubated on ice with 1 µg/ml Alexa Fluor 594-conjugated Cholera toxin B subunit (CtxB) for 30 min to label the plasma membrane. Cells were then washed twice with complete medium and left at 37°C to internalize the probe for a further 2 h followed by 3 washes in ice cold complete medium supplemented with 1 % BSA to remove extracellular label. Coverslips were mounted in Vectashield (VectorLabs) prior to visualization. All images were taken on a Zeiss Axioskop 2 microscope fitted with a Retiga CCD camera and Metamorph imaging suite.

Electron Microscopy

Cells were harvested (1 x 10^7 per cell type), washed three times with PBS and fixed in PBS containing 2 % glutaraldehyde and 20 mM Hepes on ice for 2 h. Cells were washed in 0.1 M cacodylate buffer containing 20 mM $CaCl_2$ and post-fixed in 1 % osmium tetroxide in 25 mM cacodylate buffer containing 1.5 % potassium ferrocyanide for 2 h on ice. Samples were dehydrated through an ethanol series, transferred to propylene oxide and embedded in Embed 800. Sections were stained with uranyl acetate/lead citrate and observed with a Hitachi 600 microscope at 75 kV.

Calcium Imaging with NAADP-AM

Cells grown on poly-D-lysine coated coverslips were loaded with 2 µM fluo-5F and 0.02 % pluronic F127 in a buffer containing 121 mM NaCl, 5.4 mM KCl, 0.8 mM $MgCl_2$, 2 mM $CaCl_2$, 6 mM $NaHCO_3$, 5.5 mM glucose, 25 mM Hepes, pH 7.4 for 30 min at room temperature, followed by a 30 min de-esterification. Fluo-5F fluorescence was monitored with a 40x oil immersion objective on a Zeiss 510 META laser scanning confocal microscope using excitation/emission wavelengths of 488/505-530 nm and the fluorescence at any time (F) normalized to the resting fluorescence (F_0). Analysis was done using ImageJ (http://rsb.info.nih.gov/ij/). NAADP-AM [6] stocks were made in anhydrous DMSO, and diluted to a final concentration of 1 µM in the above buffer just prior to the addition. Where indicated cells were pre-incubated with 1 nM NAADP-AM for 30 min, or 3 µM bafilomycin A1 for 60 min prior to the experiment. ATP (100 µM) was added at the end of each experiment to assess cell viability.

Calcium Imaging with Bafilomycin A1 or GPN

Cells grown on poly-D-lysine coated coverslips were loaded with 2 µM fura-2 AM and 0.02% pluronic F127 for 30 min at room temperature, followed by a 30 min de-esterification period. Imaging was performed on an inverted Olympus IX71 microscope, using OptoFluor software (CAIRN). Fura-2 was excited using 340 and 380 nm, and the emission was collected using a 480-540nm bandpass filter. The ratio 340/380 was calculated, and normalised to the baseline ratio at the beginning of the experiment. Ca^{2+} release from lysosomes was examined by incubation with 3 µM bafilomycin A1 or 200 µM GPN (Glycyl-L-phenylalanine 2-naphthylamide) and fluorescence monitored for 15 min or 5 min, respectively, after addition of compounds. Cells showing a change in ratio above 1.2 of the normalised baseline were considered as responding cells.

Lysosomal pH Measurements

Cells grown on poly-D-Lysine coated coverslips, were loaded with 0.5 mg/ml of LysoSensor Yellow/Blue dextran (10,000 MW, Invitrogen) in medium and incubated for 12 h. Dextrans were

washed off, and cells were further incubated for 24 h in tet-containing medium to induce the expression of mCherry.*Sp*TPCs and to chase the dextrans into lysosomes. Lysosomal fluorescence was monitored on an inverted Olympus IX-71 fluorescence microscope, and image acquisition was performed using OptoFluor software (CAIRN). Image pairs were taken at 340 and 380 nm excitation, and the emission was measured at 450 and 520 nm, using an Optosplit II emission image splitter (CAIRN). For analysis, the ratio of 340 excitation / 450 emission over 380 excitation / 520 emission was calculated [7]. Image acquisition was performed in imaging buffer as above and pictures of five different regions were taken per coverslip. ImageJ was used to analyse the images.

Flash Photolysis Experiments

Cells grown on poly-D-lysine coated coverslips were mounted in a chamber with a buffer containing 121 mM NaCl, 5.4 mM KCl, 0.8 mM $MgCl_2$, 2 mM $CaCl_2$, 6 mM $NaHCO_3$, 5.5 mM D-Glucose, 25 mM Hepes, pH 7.4. Cells were microinjected with a solution containing 1 mM fluo-3 plus 1 mM caged IP_3 using an Eppendorf Injectman system. Fluo-3 was monitored with a 63x oil immersion objective on a Zeiss 510 META laser scanning confocal microscope using excitation/emission wavelengths of 488/>505 nm and the fluorescence at any time (F) normalized to the resting fluorescence (F_0). After a 20 min recovery period, photolysis was initiated with pulses of 351 plus 364 nm light from a Coherent UV laser at the power and times indicated in the figure (the power was controlled via an AOTF).

Patch Clamp

Cells grown on poly-D-lysine coated coverslips were loaded with 2 µM fura-2-AM in 0.03 % pluronic F127 for 1 h at 37 ºC and infused with 100 nM NAADP through a patch pipette in the whole-cell configuration. The intracellular solution used contained: 140 mM KCl, 1 mM $MgCl_2$, 10 mM Hepes and 1 mM fura-2, pH 7.1. The bath was a physiological salt solution as in the calcium imaging experiments with NAADP-AM. Where indicated 200 µg/ml heparin or 1 µM bafilomycin A1 were added to the intracellular solution. Whole-cell voltage clamp was made at room temperature with a multiclamp 700B amplifier (Molecular Devices). Patch pipettes were pulled from borosilicate glass capillaries (1.5 mm outer diameter, WPI) on a horizontal puller (P97, Sutter Instruments). Cells were voltage clamped at -40 mV. $[Ca^{2+}]_i$ changes were measured by dual-wavelength (340 and 380 nm) excitation spectrofluorimetry, using a photometric-based system (Photon Technologies International, Princeton, NJ) to capture the emitted fluorescence at 510 nm. All experiments were performed at room temperature.

Data Analysis

Data are presented as the mean ± SEM of n preparations. Data were statistically analyzed using a Student's t test (for two means), an analysis of variance followed by a Dunnett's post-test (for multiple means against a control), an analysis of variance followed by a Tukey's post-test (for multiple means), or a non-parametric Kruskal-Wallis test followed by a Dunn's post-test (for samples not passing a normality test). P values are summarized as ns (>0.05), * (<0.05), ** (<0.01) or *** (<0.001).

Supplemental References

1. Piper, R.C., and Luzio, J.P. (2004). CUPpling calcium to lysosomal biogenesis. Trends Cell Biol *14*, 471-473.
2. Luzio, J.P., Bright, N.A., and Pryor, P.R. (2007). The role of calcium and other ions in sorting and delivery in the late endocytic pathway. Biochem Soc Trans *35*, 1088-1091.
3. Davis, L.C., Morgan, A.J., Ruas, M., Wong, J.L., Graeff, R.M., Poustka, A.J., Lee, H.C., Wessel, G.M., Parrington, J., and Galione, A. (2008). Ca^{2+} signaling occurs via second messenger release from intraorganelle synthesis sites. Curr Biol *18*, 1612-1618.
4. Calcraft, P.J., Ruas, M., Pan, Z., Cheng, X., Arredouani, A., Hao, X., Tang, J., Rietdorf, K., Teboul, L., Chuang, K.T., et al. (2009). NAADP mobilizes calcium from acidic organelles through two-pore channels. Nature *459*, 596-600.
5. Morgan, A.J., Churchill, G.C., Masgrau, R., Ruas, M., Davis, L.C., Billington, R.A., Patel, S., Yamasaki, M., Thomas, J.M., Genazzani, A.A., et al. (2005). Methods in cyclic ADP-ribose and NAADP research. In Methods in Calcium Signalling, J.W.P. Jr., ed. (CRC Press), pp. 265–334.
6. Parkesh, R., Lewis, A.M., Aley, P.K., Arredouani, A., Rossi, S., Tavares, R., Vasudevan, S.R., Rosen, D., Galione, A., Dowden, J., et al. (2008). Cell-permeant NAADP: a novel chemical tool enabling the study of Ca^{2+} signalling in intact cells. Cell Calcium *43*, 531-538.
7. Diwu, Z., Chen, C.S., Zhang, C., Klaubert, D.H., and Haugland, R.P. (1999). A novel acidotropic pH indicator and its potential application in labeling acidic organelles of live cells. Chem Biol *6*, 411-418.
